# Metabolic Insight Into the Neuroprotective Effect of Tao-He-Cheng-Qi (THCQ) Decoction on ICH Rats Using Untargeted Metabolomics

**DOI:** 10.3389/fphar.2021.636457

**Published:** 2021-05-03

**Authors:** Rui-Pei Yang, Da-Ke Cai, Yu-Xing Chen, Hai-Ning Gang, Mei Wei, De-Quan Zhu, Su-Mei Li, Jiu-Mei Yang, Si-Ni Luo, Xiao-Li Bi, Dong-Mei Sun

**Affiliations:** ^1^ Fifth Clinical Medical School, Guangzhou University of Chinese Medicine, Guangzhou, China; ^2^ Guangdong Provincial Key Laboratory of Research and Development in Traditional Chinese Medicine, Guangzhou, China; ^3^ Guangdong Yifang Pharmaceutical Co., Ltd. Foshan, China; ^4^ Guangdong Second Traditional Chinese Medicine Hospital (Guangdong Province Engineering Technology Research Institute of T. C. M), Guangzhou, China

**Keywords:** intracerebral hemorrhage, traditional Chinese medicine, Tao-He-Cheng-Qi decoction, metabolomics, secondary brain injury

## Abstract

Tao-He-Cheng-Qi decoction (THCQ) is an effective traditional Chinese medicine used to treat intracerebral hemorrhage (ICH). This study was performed to investigate the possible neuroprotective effect of THCQ decoction on secondary brain damage in rats with intracerebral hemorrhage and to elucidate the potential mechanism based on a metabolomics approach. Sprague-Dawley (SD) rats were randomly divided into five groups: the sham group, collagenase-induced ICH model group, THCQ low-dose (THCQ-L)-treated group, THCQ moderate-dose (THCQ-M)-treated group and THCQ high-dose (THCQ-H)-treated group. Following 3 days of treatment, behavioral changes and histopathological lesions in the brain were estimated. Untargeted metabolomics analysis with multivariate statistics was performed by using ultrahigh-performance liquid chromatography–mass spectrometry (UPLC-Q-Exactive Orbitrap MS). THCQ treatment at two dosages (5.64 and 11.27 g/kg·d) remarkably improved behavior (*p* < 0.05), brain water content (BMC) and hemorheology (*p* < 0.05) and improved brain nerve tissue pathology and inflammatory infiltration in ICH rats. Moreover, a metabolomic analysis demonstrated that the serum metabolic profiles of ICH patients were significantly different between the sham group and the ICH-induced model group. Twenty-seven biomarkers were identified that potentially predict the clinical benefits of THCQ decoction. Of these, 4 biomarkers were found to be THCQ-H group-specific, while others were shared between two clusters. These metabolites are mainly involved in amino acid metabolism and glutamate-mediated cell excitotoxicity, lipid metabolism-mediated oxidative stress, and mitochondrial dysfunction caused by energy metabolism disorders. In addition, a correlation analysis showed that the behavioral scores, brain water content and hemorheology were correlated with levels of serum metabolites derived from amino acid and lipid metabolism. In conclusion, the results indicate that THCQ decoction significantly attenuates ICH-induced secondary brain injury, which could be mediated by improving metabolic disorders in cerebral hemorrhage rats.

## Introduction

Intracerebral hemorrhage (ICH) is primarily classified clinically as spontaneous (e.g., haemorrhagic stroke) or traumatic, and it accounts for 9–27% of all strokes worldwide (Qureshi et al., 2009; Shah et al., 2019; Quiñones-Ossa et al., 2020; Xu et al., 2020). When it is caused by non-traumatic or spontaneous ICH, the physician must consider different etiologies (Steiner et al., 2014). ICH-induced brain injury is conventionally divided into 2 subtypes: primary brain injury and secondary brain injury. Comparatively, in primary brain injury, brain tissue is impaired by mechanical impact initiated by the hemorrhage and growth of the hematoma in the early phase of ICH (Zhou et al., 2014), while in secondary brain injury, the brain tissue is further and progressively damaged by red blood cell debris and other clot components (Chen et al., 2015; Zhao et al., 2019). Emerging studies have revealed that the pathogenesis of secondary brain injury (SBI) involves multiple molecular mechanisms and processes, such as apoptosis, inflammatory cascades, ischemia, blood–brain barrier disruption, and brain edema formation (Beez et al., 2017; Gao et al., 2018). Currently, multiple treatments for ICH are available, including surgery, the control of intracranial hypertension and blood pressure, the alleviation of cerebral edema, supportive care, and rehabilitation. However, symptomatic treatment is still a common remedy for ICH patients, but recovery post-ICH is not adequate due to the irreversible neurological damage caused by the SBI (Elliott and Smith, 2010). Therefore, since ICH patients still poorly benefit from current interventions, alternative treatments for ICH are needed (Morgenstern et al., 2010).

Historically, traditional Chinese medicine (TCM) prescriptions have long been used in the treatment of acute ICH. The Liangxue Tongyu formula (LXTYF), which is a derivative prescription from “Xi Jiao Di Huang Tang” (mentioned in the ancient traditional Chinese medicine book “Bei Ji Qian Jing Yao Fang” in AD 652), is frequently used as clinical practice adjuvant therapy in China (Li et al., 2015). As an alternative medicine, LXTYF is an example of a TCM formula used for treating ICH according to a specific theory. Furthermore, THCQ is a well-known formulation that has the function of clearing heat and resolving stasis, as documented in Shang Han Za Bing Lun (a book on TCM for febrile and miscellaneous diseases written in the Han Dynasty, AD 200-210), with ICH treatment prescriptions (Zhang et al., 2016). Recently, this theory has been partially validated in clinical practice and scientific research. Twenty-five studies and 15 TCM formulas have been proven to have benefits for ICH, including “Shengyu” decoction, Huayu capsules, Quyu Tongfu decoction, and Longxuejie capsules, by potentially showing neuroprotective effects in terms of reducing brain water content and improving blood-brain barrier permeability (Yang et al., 2016). THCQ decoction is consistently classified as a cardio-cerebrovascular prescription (Ranran et al., 2020). Its *jun* drug (monarch herb), peach kernel, is one of the most commonly used herbal medicines for the treatment of acute cerebral hemorrhage in clinical practice (Li et al., 2015). The other *jun* drug (monarch herb) drug, rhubarb, has obvious effects in cerebral hemorrhage models *in vivo* and *in vitro* (Zhou et al., 2011; Wang et al., 2016). It shows prospects for treating ICH according to traditional theory and evidence from current research (Min-jie et al., 2018). However, it remains uncertain whether THCQ decoction could be beneficial for ICH patients by regulating multiple systems. In our research, collagenase-induced ICH was applied to evaluate the efficacy of THCQ decoction. Moreover, a systemic level method was employed to explore the neuroprotective mechanism.

In the last decade, metabolomics belongs to the “omics” sciences and has been well established and is advancing our comprehension of the global effects of studied objects at the systemic level (Wang et al., 2020). Since a TCM formulation is a complicated mixture of compounds, it is not easy to identify the regulation targets. Serum metabolomics will solve the identification problem at the endogenous metabolic level to reveal the mechanism of ICH treatment. The THCQ formulation can be studied via multiple small molecules and related pathways.

In this study, untargeted metabolomics based on the UPLC-Q-Exactive Orbitrap MS system and multivariate statistical analysis was performed, and it was found that the therapeutic effect of THCQ decoction on an ICH rat model was related to metabolic changes related to blood cytotoxicity and oxidative stress. The multivariate analysis revealed important information related to regulation between the THCQ decoction and Prominent metabolites KEGG Pathway terms enriched in the metabolic pathways. Collectively,the sampling method used for serum metabolomics made it applicable to clinical practice, and metabolomics data from the treatment of ICH with THCQ decoction will provide novel insights into clinical applications as well.

## Materials and Methods

### Chemicals and Reagents

HPLC-grade acetonitrile and formic acid were acquired from Merck KGaA (Merck, Darmstadt, Germany). Deionized water was prepared using a Millipore water purification system (Millipore, Merck, Darmstadt, Germany). Bacterial collagenase type VII-S was procured from Sigma Aldrich (St. Louis, MO, United States). Chloral hydrate from Macklin (Shanghai, China) was used to anesthetize rats. Paraformaldehyde was purchased from Shenggong Biotech (Shanghai, China).

### Preparation of THCQ Decoction

Tao-He-Cheng-Qi (THCQ) decoction is composed of 5 kinds of active materials, including Semen Persicae (the seeds of *Prunus persica* L.), Rheum officinale (the herb of *Rheumpalmatum* L., *Rheum tanguticum* Maxim. ex Balf. or *Rheum offcihale* Baill.), Ramulus Cinnamoml, Herba Licorice, and Mirabilite (Natrii sulfas), at a fixed ratio of 1:4:2:2:2. All crude drugs were provided by Guangdong Yifang Pharmaceutical Co., Ltd. (Foshan, China) and authenticated by Prof. D.M. Sun (Guangzhou University of Chinese Medicine, Guangzhou, China) based on the botanical traits recorded in the Chinese Flora (http://www.efloras.org/index.aspx). All voucher specimens (Supplementary Table S1 and Supplementary Figure S1) were deposited at the Department of Traditional Chinese Medicine Analysis Laboratory, Guangdong Second Traditional Chinese Medicine Hospital (Guangdong Province Engineering Technology Research Institute of TCM), Guangzhou, China. For preparation of the THCQ decoction, the mixed crude drugs except sodium sulfate were soaked in purified water at room temperature (28°C) for 0.75 h. The drugs were boiled in a 10-fold volume of water (1:10, w/v) for 1.0 h before filtration. Then, sodium sulfate was added to the decoction, which was brought to a boil before filtration and concentration under vacuum. The concentrated water decoction was freeze-dried, and the extraction rate was 10%. The THCQ extract was then stored at -20°C and fully suspended in water before use.

### Chemical Analysis of THCQ Decoction

The lyophilized powder of THCQ decoction was then redissolved in 10 ml of double-distilled water. Samples were filtered with a 0.22 µm membrane before UPLC-QTOF-MSE (Sciex, United States) analysis. Separation was achieved on an HSS T3 column (150 mm × 2.1 mm, 2.5 µm; Waters). Analytes were separated using a Waters HSS T3 column (2.1 mm × 100 mm × 1.8 µm) with a column temperature of 40°C and a flow rate of 0.35 ml/min. Mobile phase A was methanol, and mobile phase B was water containing 2 mmol/L ammonium acetate and 0.05% formic acid. A gradient elution program was applied (0–5 min, 85% B; 5–14 min, 70% B; 14–23 min, 65% B; 23–28 min, 58% B; 28–42 min, 50% B; 42–56 min, 34% B; 56–72 min, 12% B; 72–76 min, 0% B). The instrument was operated under negative ion mode to acquire data in the m/z range of 50–1,500 Da. The curtain gas, ion source gas 1, ion source gas 2 and collision gas (all high-purity nitrogen) were set at 241 kPa, 241 kPa, 345 kPa and 0.345 MPa, (35, 50, 50 psi/145), respectively. The spray voltage floating was set at 5,500 V and −4500 V for positive and negative ion modes, respectively. and the capillary temperature was 500°C. By following this approach, the 20-compound data analysis was carried out by comparing the high-resolution MS data with reported data. The full details for the 20 compounds are presented in the Supplementary material online, Supplementary Tables S1, S2, and Supplementary Figure S2.).

### Animals and Treatment

Animal experiments were conducted according to the recommendations of the National Institutes of Health Guide for the Care and Use of Laboratory Animals. The protocol for the animal experiments was approved by the Experimental Animal Ethics Committee of Guangdong Second Traditional Chinese Medicine Hospital (Guangdong Province Engineering Technology Research Institute of TCM) (application number 048721). Male Sprague-Dawley (SD) rats (8 weeks old) were obtained from the Guangdong Medical Laboratory Animal Center (Guangzhou, China, certificate No. SCXK(Yue)2018-0002) and kept at the Institute for Pharmacology of Traditional Chinese Medicine in specific pathogen-free conditions at the Experimental Animal Center of the Guangdong Second Traditional Chinese Medicine Hospital (Guangdong Province Engineering Technology Research Institute of TCM, Guangzhou, China), having free access to food and water ad libitum. Adult SD, maintained on a 12 h-12 h light-dark cycle ICH rats were induced by stereotaxic infusion of bacterial collagenase VII-S (0.25 U in 1.0 μl sterile saline, Sigma-Aldrich), which were injected into the right striatum over a 10-min period (Feng et al., 2015; Zeng et al., 2017) and then randomly divided into four groups (ICH group, ICH + THCQ-L group, ICH + THCQ-M group, and ICH + THCQ-H group, n = 6 per group). Meanwhile, age-matched sham-operated rats were treated via the same method except that they were administered 1 μl sterile normal saline into the right striatum (control group, n = 6 per group) (Zeng et al., 2017). Rats in the ICH + THCQ-L group, ICH + THCQ-M group and ICH + THCQ-H group were orally administered THCQ decoction at dosages of 2.82 g/kg, 5.64 g/kg and 11.27 g/kg (equal to the clinical dose, calculated by the weight of the crude drugs), respectively, once daily, for 3 consecutive days after the ICH model. The sham group and ICH group of animals received an equal volume of distilled water.

### Behavioral Testing

Behavioral tests were assessed with Modified neurological severity scores (*mNSS*) (Chen et al., 2001; Ren et al., 2018) at 6, 24 and 72 h after ICH by an investigator who was blinded to the experimental groups. Scores range from 0 to 100 with higher scores indicating a more severe neurological injury, which are defined as follows: a score of 13 to 18 indicates severe injury, 7 to 12 indicates moderate injury, and 1 to 6 indicates mild injury. Rats were assessed 1 point if they failed to perform a task.

### Hemorheological Tests

Two milliliters blood samples were collected into Vacutainer tubes (BD, Franklin Lakes, NJ, United States) containing ethylenediaminetetraacetic acid (EDTA, an anticoagulant), after which 0.8 ml whole blood samples were prepared for whole blood viscosity (WBV) examination. Plasma was obtained by centrifugation of the remaining blood at 1000 g for 10 min, after which 0.5 ml of plasma was used for plasma viscosity (PV) examination. The automated hematology analytical instrument LBY-N7500A (Pulisheng Company, Beijing, China) was used for the measurement of WBV and PV at 37.0  ±  0.5°C (Shu et al., 2018). For WBV, measurements were made at three shear rates: 5, 60 and 150 s^−1^. PV was measured at a shear rate of 60 s^−1^. In addition to measuring RBC deformability, the automated hematology analyzer system was also utilized to determine RBC aggregation (Tripette et al., 2009).

### Histopathology Assessment

All rats were deeply anesthetized with an intraperitoneal injection of 10% chloral hydrate (3 ml/kg). After blood collection, rat brains were fixed by transcardial perfusion with saline, followed by perfusion and immersion in 4% paraformaldehyde (PFA) at 4°C overnight, and the brain was embedded in paraffin. The cerebral tissues were paraffin-embedded, and cross sections (2 µm) were cut (Wu et al., 2018a). The rat tissue sections were stained with hematoxylin and eosin (H&E) for histopathological examination, and images were acquired by DP2-BSW acquisition software (Olympus) (Zhang et al., 2020).

### Serum Metabolomics Study

**Sample Preparation** Rats were euthanized 72 h post-surgery.Blood samples from the abdominal aorta were collected in vacutainer tubes and serum obtained by centrifugation 10 min at 3,000 rpm. All tubes were then centrifuged at 13,000 rpm at 4°C for 10 min, and supernatants were collected and immediately stored at −80°C for metabolic analyses.100-μl aliquot of serum samples were transferred to a clean 1.5 ml Eppendorf tube on ice, and 400 μl of cold methanol-acetonitrile (3:1, vol/vol) was added, mixed well and vortexed for 30 s. Samples were then spun at 4°C for 10 min at 13,000 rpm, and the supernatant was transferred to 200 μl glass vial inserts and subjected to UPLC-Q-Orbitrap MS.A quality control (QC) sample was created by mixing equal volumes of each sample (10 µL) and treated as described above. QC samples were injected after every eight samples throughout the run (Liu et al., 2020).

**Analysis Conditions** An aliquot of 3 μl was injected into an Acquity UPLC HSS T3 column (1.8 µm, 2.1 × 100 mm) equipped with a VanGuard^TM^ pre-Column (1.8 µm, 2.1 × 5 mm) (Waters, MA, United States) maintained at 30°C using a Thermo UltiMate3000 UHPLC system (Thermo Fisher, MA, United States) with a ZprayTMESI source for chromatographic separation. The mobile phases consisted of water containing 0.1% formic acid (A) and acetonitrile (B). The gradient elution was performed as follows at a flow rate of 0.3 ml/min for favorable separation: 0.0–5.0 min, 95%–95% C; 5.0–10.0 min, 95%–5% A; 10.0–15.0 min, 15%–5% A; 15.0–24.5 min, 5%–95% A; 24.5–28.0 min, 95%–95% A. The Q-Exactive Focus Orbitrap mass spectrometer was operated in both negative and positive ion modes. The ion source conditions were set as follows: evaporation temperature, 350°C; capillary temperature, 320°C; sheath gas, 35 Arb; spray voltage, 2.8 kV; auxiliary gas, 10 Arb; and S-lens RF, 50. The other parameters were set as follows: grade I, full scan (±); resolution, 70,000; maximum TT, 100 ms; AGC target, 1e^6^; scan range, 70-1,000 m/z (Wang et al., 2020).

**Data Processing** The metabolite annotation of the LC-MS data was performed with the Compound Discoverer program 3.1 (Thermo Fisher Scientific) with reference to the mzCloud database (www.mzCloud.org). The identified ions were queried in the mzCloud database (www.mzCloud.org), the Human Metabolome Database (www.hmdb.ca), Metlin (metlin.scripps.edu), MassBank (www.massbank.jp), and the Kyoto Encyclopedia of Genes and Genomes (http://www.genome.jp/kegg/ligand.html) (Liu et al., 2017; Huang et al., 2019; Ju et al., 2020). Principal component analysis (PCA) and partial least squares discriminant analysis (PLS-DA) were performed with MetaboAnalyst 4.02 (http://www.metaboanalyst.ca/). The data were statistically processed using SPSS 24.0 (SPSS Inc., Chicago, IL, United States). Data are expressed as the mean ± standard deviation (SD), and Student's t-test or one-way ANOVA was used for comparisons between groups. Metabolites with both multidimensional statistical analysis VIP > 1 and univariate statistical analysis *p* value < 0.05 were selected as metabolites with significant differences. Statistical tests were performed using GraphPad Prism 8.4.2 software (GraphPad Software Inc., San Diego, United States) to determine the changes in potential biomarkers in the four groups. MetaboAnalyst (http://www.metaboanalyst.ca/) was used for the correlation analysis, metabolic pathway analysis, heat mapping and hierarchical cluster analysis.

## Results

### THCQ Attenuates Brain Injury and Improves Outcomes After ICH

**FIGURE 1:**
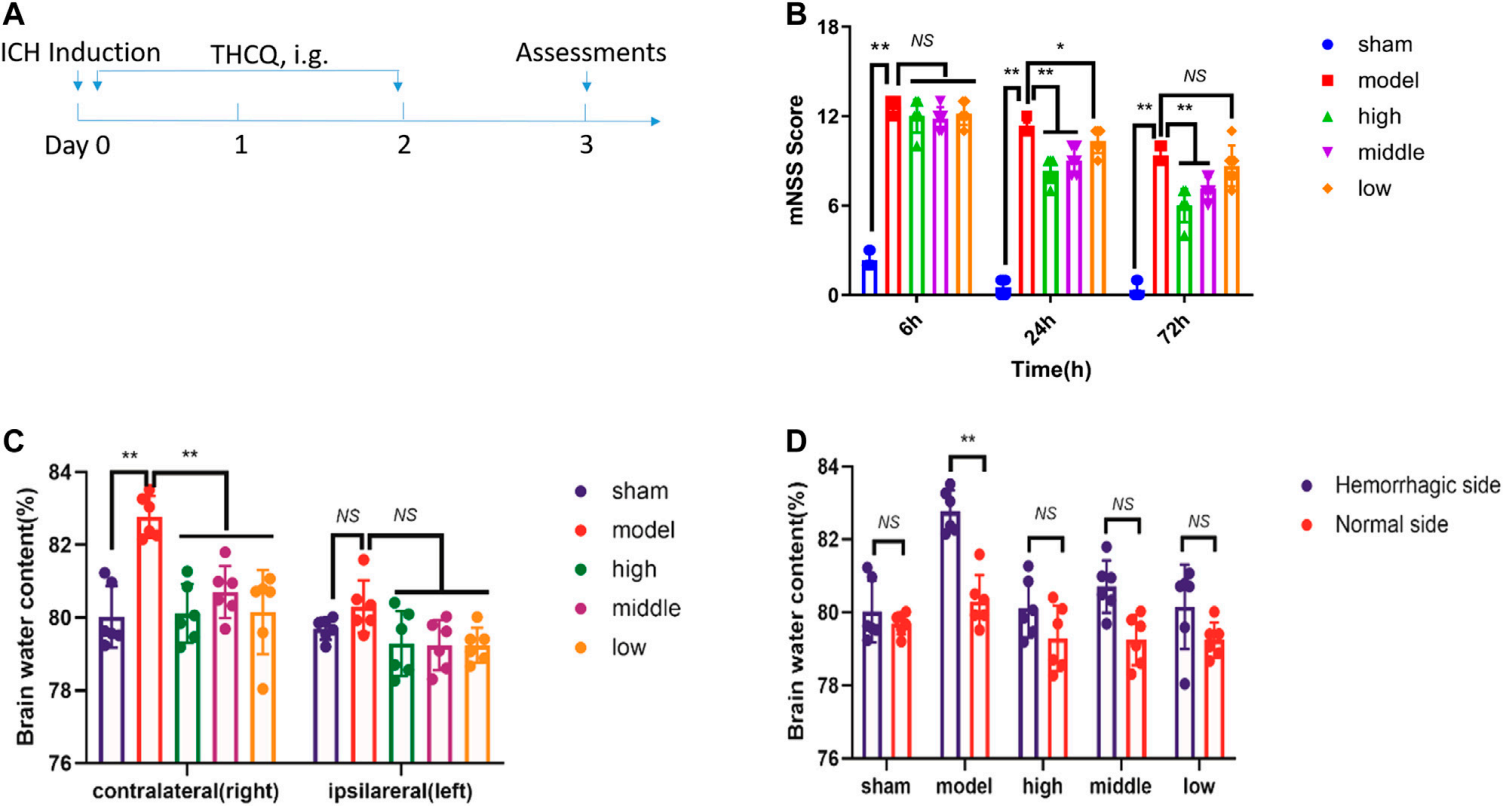
THCQ attenuates brain injury and improves long-term outcomes after ICH. ICH was induced in SD rats by injection of collagenase. Flow chart illustrating THCQ administration and the experimental design. Rats received daily intraperitoneal (IG) injections of THCQ (28.2, 56.4 and 112.7 mg/kg) or an equal volume of phosphate-buffered saline (PBS) vehicle for 3 consecutive days starting immediately after ICH induction **(A)**. Neurological tests were performed to evaluate the motor, sensory, and balance functions in mice receiving vehicle or THCQ at 6 h, 24 h, and 72 h after injection of collagenase (right) **(B)**. Similarly, THCQ treatment at doses of 28.2, 56.4 and 112.7 mg/kg markedly alleviated the histological changes evaluated via the BWC **(C, D)** (n = 6 rats/group) measured by the dry/wet weight method at 72 h after ICH. n = 6 per group. Data are presented as the mean ± SD. *p < 0.05, **p < 0.01.

To establish whether THCQ decoction decreases brain injury after ICH, we compared neurodeficits and brain water content in ICH rats receiving THCQ decoction or phosphate-buffered saline vehicle. And adopted a collagenase-induced ICH model Rats received THCQ (28.2, 56.4 and 112.7 mg/kg) or vehicle for 3 consecutive days starting immediately after ICH induction (Figure 1A). Neurological deficits, crudely assessed using the *mNSS* at 6 hours, 24 hours and 72 hours after ICH. At 3 days after ICH onset, brain water content was determined using the wet/dry method. Compared with vehicle recipients, THCQ-treated rats displayed significantly reduced in neurodeficits and brain water content after ICH (Figures 1B–D). In addition, the brain water content of the right and left cerebral hemispheres was analyzed (Figure 1). The right cerebral hemisphere brain water content in untreated ICH animals (model group; n = 6) was significantly higher than that in control animals (sham group). ICH animals treated with THCQ had significantly lower right cerebral hemisphere brain water content (ICH+THCQ) than untreated ICH animals (sham group) (*p* < 0.05). There was no observable brain water content loss of the contralateral cerebral hemisphere (left cerebral hemisphere) in all treated groups compared with the control group (sham group) (*p* > 0.05). [However, brain water content in non-injured hemispheres did not differ among treatment group (*p* > 0.05)].

### Hemorheology Indexes

**FIGURE 2:**
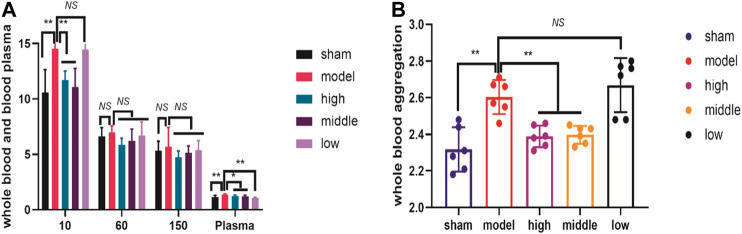
THCQ attenuates brain injury and improves hemorheology indexes after intracerebral hemorrhage (ICH). ICH was induced in SD rats by injection of collagenase. Hemorheology tests were performed to evaluate the reduction of the whole blood viscosity at low shear and the plasma viscosity in rats receiving vehicle or THCQ at day 3 after injection of collagenase (right) **(A)**. Hemorheology tests were performed to evaluate the extent of RBC aggregation (aggregation index) in rats receiving vehicle or THCQ at day 3 after injection of collagenase (right) **(B)**.

We measured the hemorheology indexes at day 3 after ICH modeling. At day 3, the plasma viscosity index was significantly lower in all dosed groups than in the model group (Figure 2A). Whole blood showed reduced viscosity at low shear (Figure 2A), and the extent of RBC aggregation (aggregation index, Figure 2B) was significantly lower in the high-dose and moderate-dose groups than in the model group. Additionally, the hemorheology indexes of the high-dose and moderate-dose groups were superior to those of the low-dose group at day 3, suggesting the benefits of the higher doses (Figure 2).

### THCQ Decoction Reduced Pathomorphological Changes in Brain Nerves in ICH Rats

**FIGURE 3:**
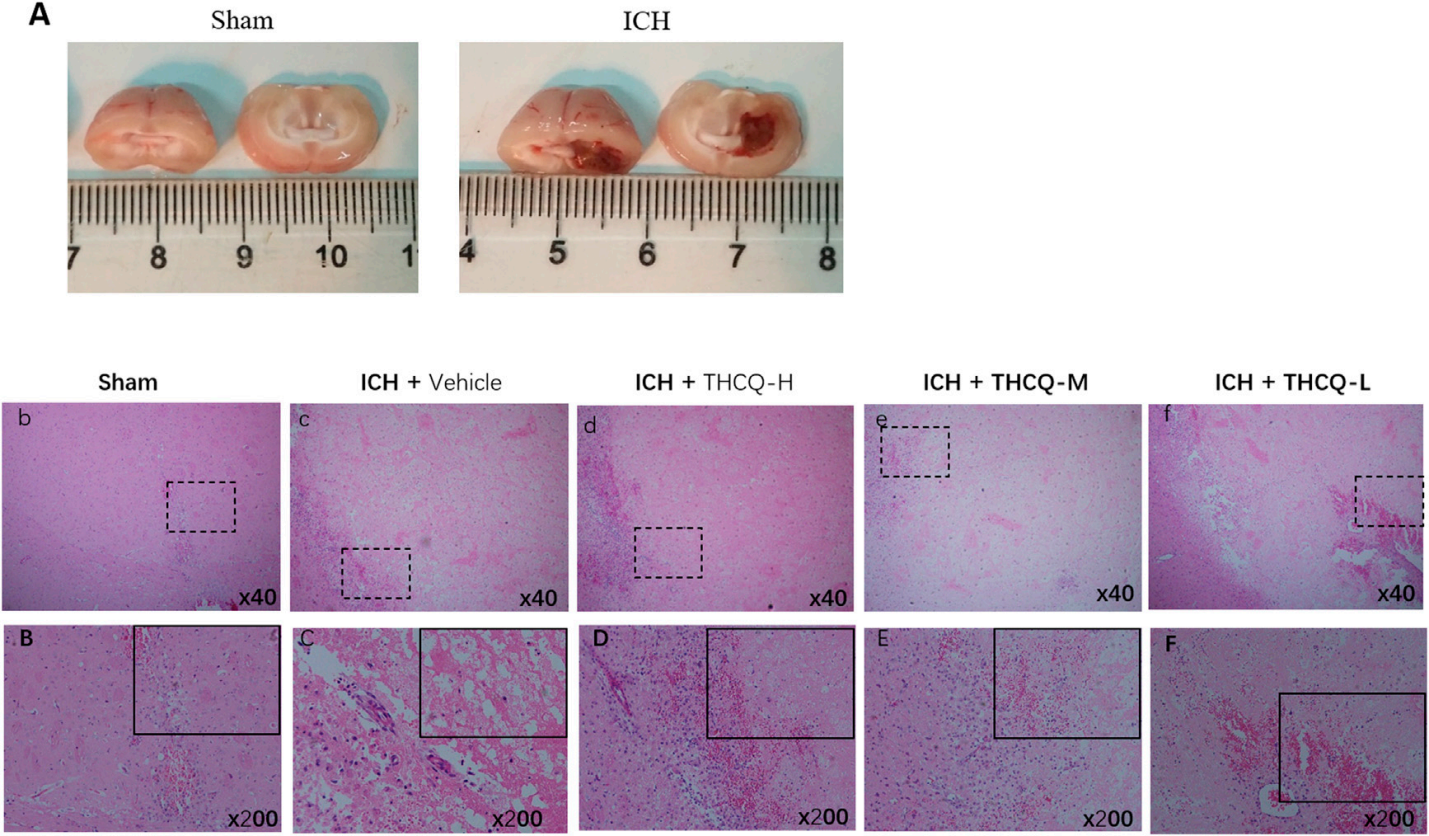
Effects of THCQ on collagenase-induced ICH. SD rats were challenged by right striatum injection of 4 μg collagenase. Brain tissues were collected 72 h after challenge with collagenase. Typical macrographs (left: sham, 72 h after operation; right: 72 h after ICH induction) **(A)**. Normal cerebral striatum **(b, B)** and striatum 72 h after collagenase injection, showing infiltration of inflammatory cells **(c, C)**, ICH + THCQ-L (28.2 mg·kg^−1^) **(d, D)**, ICH + THCQ-M (56.4 mg·kg^−1^) **(e, E)** and ICH + THCQ-H (112.7 mg·kg^−1^) **(f, F)**. The histological morphology and pathology results showed that treatment with THCQ alleviated ICH-induced pathological changes.

Compared with the control group (sham), histopathological examination of the striatum after ICH revealed neuronal cells that were loosely arranged; the gaps were enlarged, some nuclei were reduced, nuclear staining was reduced, and pyknosis, deep staining, and cytoplasmic vacuolation were obvious. The pathological damage was serious, as neutrophils has been recruited to the vesicle cavity in large numbers, and inflammatory infiltration of lymphocytes was obvious. However, these collagenase-induced pathological changes were significantly attenuated by treatment with THCQ (28.2, 56.4 and 112.7 mg·kg^−1^) (Figure 3).

### Metabolomics Analysis

#### Metabolic Profiling of Serum

We tested the effects of a number of different conditions on the mobile phases. The best results were obtained using aqueous 0.1% formic acid solution (mobile phase A) and acetonitrile (mobile phase B); these were therefore selected for further analyses.

**FIGURE 4:**
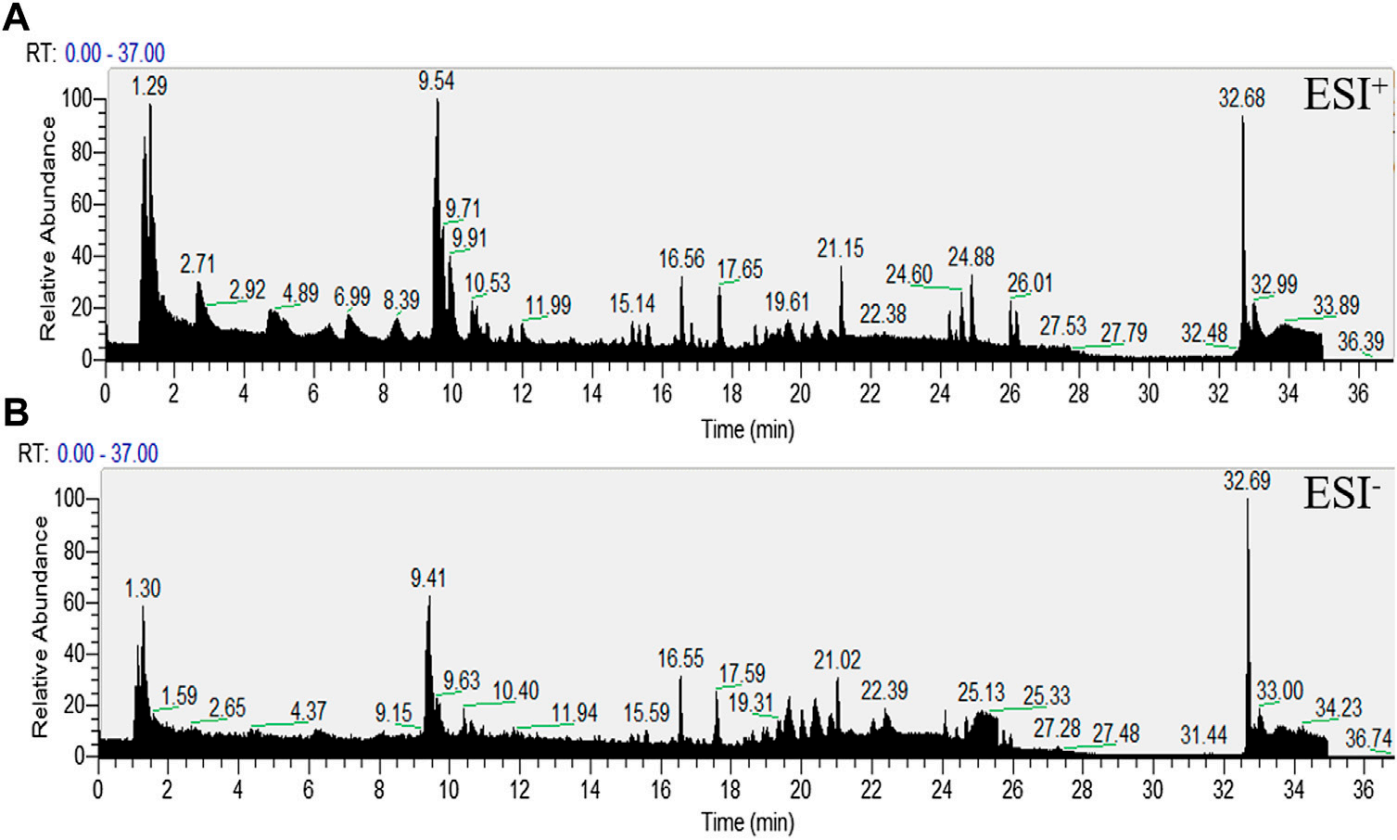
Representative total ion chromatograms (TIC) obtained in serum in ESI+ and ESI- modes.

The representative LC-MS total ion current chromatograms (TIC) obtained in negative and positive ESI modes are shown in Figure 4.

The reproducibility and stability of the LC-MS method were fundamental to the metabolomics analysis and were assessed by using quality control (QC) samples. The six QC samples were clustered close together in the PCA score plot, indicating high repeatability and good instrument stability throughout the LC-MS analysis. Therefore, the developed method had good repeatability and stability. (Supplementary Figure S3).

#### Multivariate Statistical Analysis

An unsupervized and comprehensive view of the PCA was constructed to explore the distribution and tendencies of the sham, ICH, ICH + THCQ-H, and ICH + THCQ- M groups. As shown in Figure 4, a good separation trend among the four groups in 3D space was analysis in both positive ion mode (ES^+^) and negative ion mode (ES^−^). This observation indicates that significant metabolic changes occurred 3 days after ICH induction. Moreover, the ICH + THCQ-H and ICH + THCQ- M groups displayed very similar metabolic phenotypes.

**FIGURE 5:**
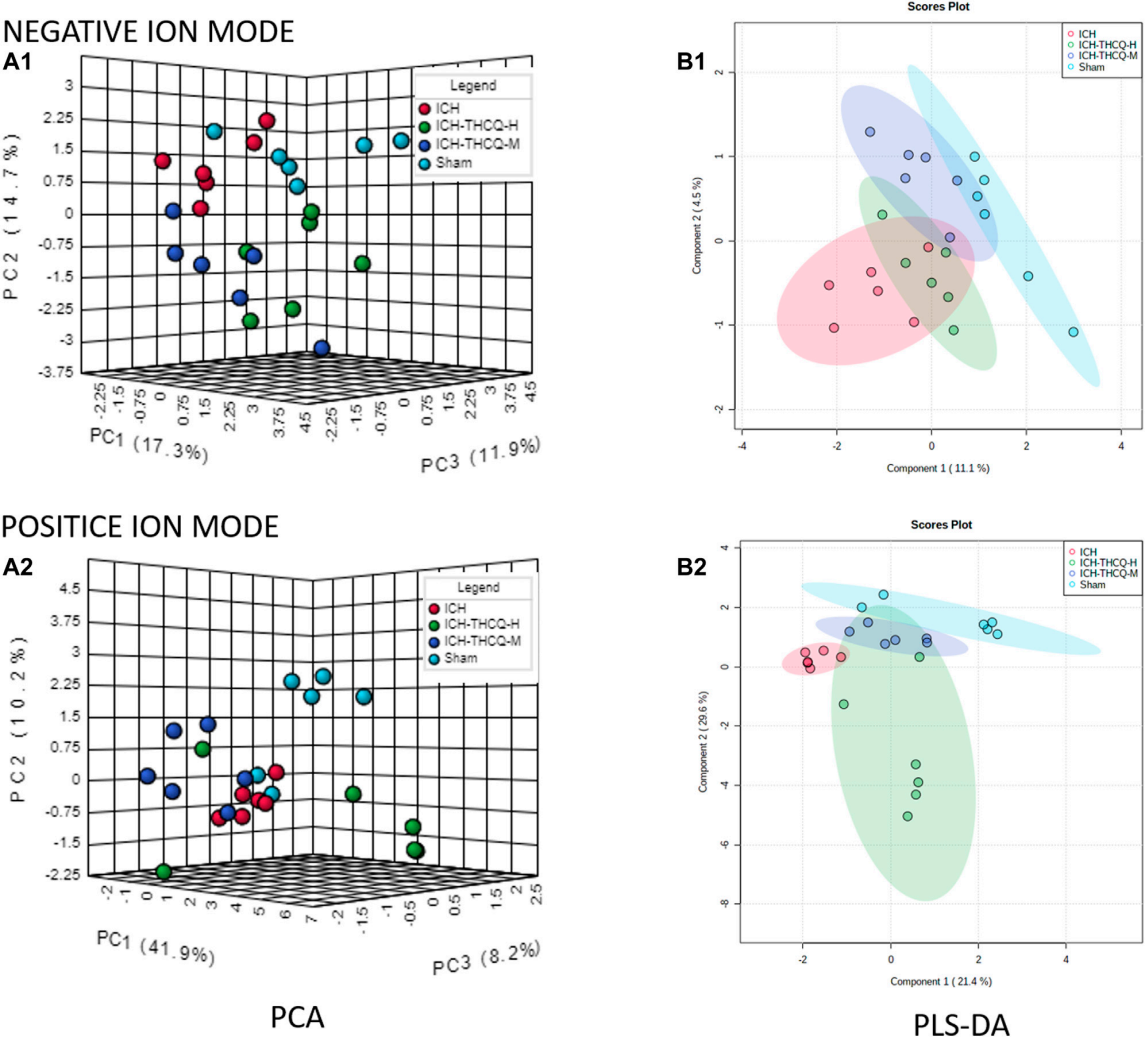
Discrimination of the serum metabolic profile among the sham, ICH, ICH + THCQ-H, and ICH + THCQ-M rats. **(A1, A2)** PCA score plots in negative and positive ion modes, respectively. **(B1, B2)** PLS-DA score plots in negative and positive ion modes, respectively, n = 6 per group.

In addition, a supervised PLS-DA analysis was used to sharpen the separation among groups and enhances recognition of these variables that contribute to categorical. Figure 5B distinct clustering of the sham, ICH and ICH + THCQ (H/M) groups was exhibited in both negative and positive ion modes, which showed that the differences between experimental groups were greater than the within-group sample differences (Figure 5). This suggests that the serum metabolic profiles after ICH induction and THCQ decoction treatment were dramatically changed.

#### Identification of Potential Biomarkers

**FIGURE 6:**
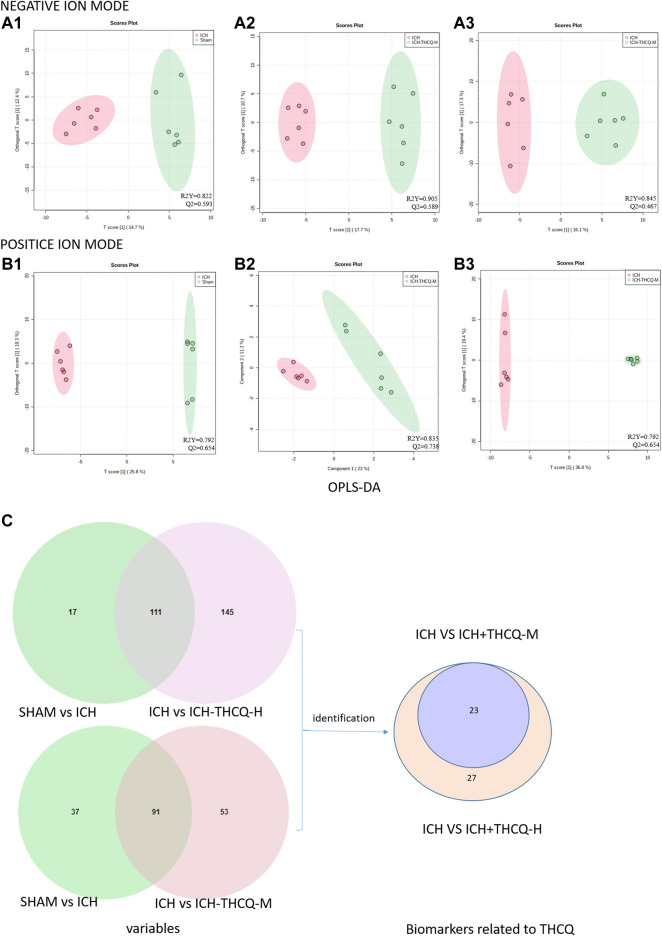
Screening of serum candidate biomarkers. (A, B) OPLS-DA score plots of the sham vs. ICH groups **(A1, B1)**, ICH vs. ICH + THCQ -H groups **(A2, B2)**, and ICH vs. ICH + THCQ -M groups **(A3, B3)** in negative and positive ion modes, respectively (n = 6 per group). **(C)** Venn diagram illustrated the overlapping and unique differential metabolites among the comparison groups. Serum levels of 23 metabolites variations in the intermediate-dose and high-dose groups were prominently recovered, while 4 other metabolites level were only regulated at high doses of THCQ decoction.

The OPLS-DA results indicated significantly changes in ions between the sham and ICH groups, ICH and ICH + THCQ-H groups, and ICH and ICH + THCQ-M groups. The OPLS-DA method is a supervised discriminant analysis statistical method that can best reflect the differences between classification groups. R2Y ≥ 0.79 and Q2 ≥ 0.46, ndicating the good quality and accurate predictive capability of the model (Figure 6). Simultaneously, the variable importance of the projection (VIP) plots of each metabolite was generated to screen the potential differential metabolites; a VIP value >1 and *p* < 0.05 according to Student’s t-test were used as the criteria. According to these criteria, 128, 256 and 144 variable ions were identified in the sham group vs. ICH group, ICH group vs. ICH + THCQ -H group, and ICH group vs. ICH + THCQ -M group, respectively. A series of matched results were then obtained after searches of online databases such as mzCloud and ChemSpider (exact mass or formula).

**FIGURE 7:**
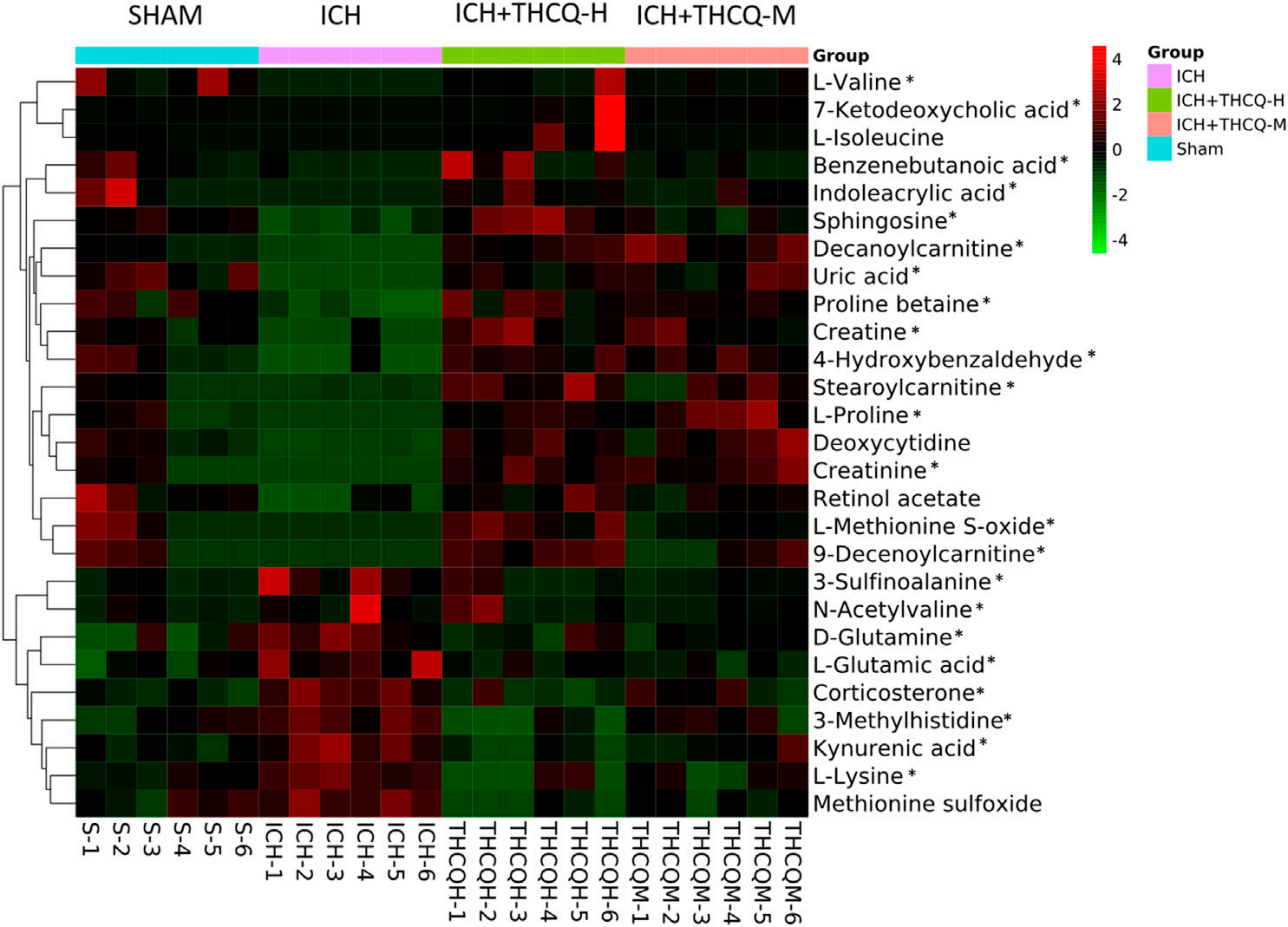
Heatmap of 27 serum metabolic candidate biomarkers in each sample. Serum levels of the 27 metabolites were striking increased by administration of THCQ decoction at a high-dose (THCQ-H) in the sham, ICH, ICH + THCQ-H, and ICH + THCQ-M groups (n = 6 per group). *Serum metabolites significantly changed by THCQ decoction administration at a medium-dose (THCQ-M).

We identified a total of 128 metabolites in serum in the ICH groups that were significantly altered compared to those in the sham group. Among these metabolites, 27 metabolites (Table 1) changed gradually at high THCQ treatment levels (VIP >1 in OPLS-DA and *p* < 0.05), tending toward the sham levels. Of the 27 target metabolites, 23 metabolites were also noticeably modulated by THCQ decoction treatment at moderate dosages. Trends with regard to the relative concentrations of the 27 potential biomarkers in the samples were visualized using a heatmap. As demonstrated in (Figure 7), 9 metabolites were upregulated in the ICH model group, and the other metabolites were downregulated. These findings suggest that THCQ decoction largely has preventive effects on metabolic disorders caused by ICH.

**TABLE 1:** Twenty-seven identified potential biomarkers regulated by Thao-He-Cheng-Qi (THCQ) decoction.

No.	Retention time (min)	Precursor ion form	Measured mass (Da)	Predicted mass (Da)	Mass error (ppm)	Identification	Molecular formula	Trend in ICH^a^	Trend in ICH + THCQ(H)^b^	Trend in ICH+ THCQ(M)^b^
1	1.159	[M + H]^+^	113.05883	113.05891	-0.74	Creatinine	C4H7N3O	↓^**^	↑^**^	↑^**^
2	1.175	[M + H]^+^	115.0634	115.06333	0.59	L-Proline	C5H9NO2	↓^*^	↑^**^	↑^**^
3	35.016	[M + H]^+^	122.03676	122.03678	-0.17	4-Hydroxybenzaldehyde	C7H6O2	↓^*^	↑^**^	↑^**^
4	1.251	[M + H]^+^	131.06946	131.06947	-0.10	Creatine	C4H9N3O2	↓^*^	↑^*^	↑^*^
5	1.225	[M + H]^+^	117.07921	117.07898	1.97	L-Valine	C5 H11 N O2	↓^**^	↑^**^	↑^**^
6	1.393	[M + H]^+^	131.09431	131.094635	-2.48	L-Isoleucine	C6H13NO2	↓^*^	↑^**^	ns
7	1.173	[M + H]^+^	143.0946	143.09463	-0.20	Proline betaine	C7H13NO2	↓^**^	↑^**^	↑^**^
8	1.258	[M + H]^+^	146.0697	146.06914	3.85	D-Glutamine	C5H10N2O3	↑^**^	↓^**^	↓^**^
9	1.018	[M + H]^+^	146.10619	146.10553	4.52	L-Lysine	C6H14N2O2	↑^**^	↓^*^	↓^*^
10	1.179	[M + H]^+^	147.05369	147.05316	3.59	L-Glutamic acid	C5H9NO4	↑^**^	↓^*^	↓^**^
11	24.418	[M - H]^-^	153.00919	153.00958	-2.57	Benzenebutanoic acid	C3H7NO4S	↑^**^	↓^**^	↓^**^
12	24.474	[M - H]^-^	159.08909	159.08954	-2.82	L-Methionine S-oxide	C7H13NO3	↑^**^	↓^*^	↓^**^
13	15.516	[M + H]^+^	164.08365	164.08373	-0.46	Methionine sulfoxide	C10H12O2	↓^**^	↑^**^	ns
14	1.417	[M + H]^+^	165.0457	165.04596	-1.57	Uric acid	C5H11NO3S	↓^*^	↑^**^	↑^**^
15	1.317	[M + H]^+^	165.04655	165.04596	3.58	3-Methylhistidine	C5H11NO3S	↑^**^	↓^**^	↓^**^
16	1.317	[M + H]^+^	168.02824	168.02834	-0.57	Indoleacrylic acid	C5H4N4O3	↓^*^	↑^**^	↑^*^
17	4.466	[M + H]^+^	169.08486	169.08513	-1.59	Kynurenic acid	C7H11N3O2	↑^*^	↓^**^	↓^*^
18	13.1	[M + H]^+^	187.0631	187.063324	-1.20	Deoxycytidine	C11H9NO2	↓^**^	↑^**^	↑^*^
19	6.101	[M + H]^+^	189.04331	189.04259	3.82	Sphingosine	C10H7NO3	↑^**^	↓^**^	↓^**^
20	1.212	[M + H]^+^	227.09026	227.090607	-1.53	9-Decenoylcarnitine	C9H13N3O4	↓^*^	↑^**^	↑^*^
21	15.558	[M + H]^+^	299.28188	299.28244	-1.87	Decanoylcarnitine	C18H37NO2	↓^**^	↑^**^	ns
22	14.763	[M + H]^+^	313.22463	313.225311	-2.17	Retinol acetate	C17H31NO4	↓^*^	↑^**^	ns
23	18.627	[M + H]^+^	315.24051	315.24096	-1.43	Corticosterone	C17 H33 N O4	↓^*^	↑^**^	↑^**^
24	24.199	[M + H]^+^	328.23991	328.240234	-0.99	7-Ketodeoxycholic acid	C22H32O2	↓^**^	↑^*^	↑^*^
25	18.698	[M + H]^+^	346.21349	346.21442	-2.68	Stearoylcarnitine	C21 H30 O4	↑^**^	↓^**^	↓^**^
26	15.624	[M + H]^+^	406.27087	406.271912	-2.56	3-Sulfinoalanine	C24H38O5	↓^*^	↑^**^	↑^*^
27	21.943	[M + H]^+^	427.36574	427.36615	-0.96	N-Acetylvaline	C25H49NO4	↓^*^	↑^**^	↑^**^

↑ upregulated; ↓ downregulated; *p < 0.05; **p < 0.01 ns, no significant difference with *p* > 0.05. ^a^ Compared with the control group. ^b^ Compared with the ICH model group.

#### Exploratory Analysis of Diversified ROC Curves

**FIGURE 8:**
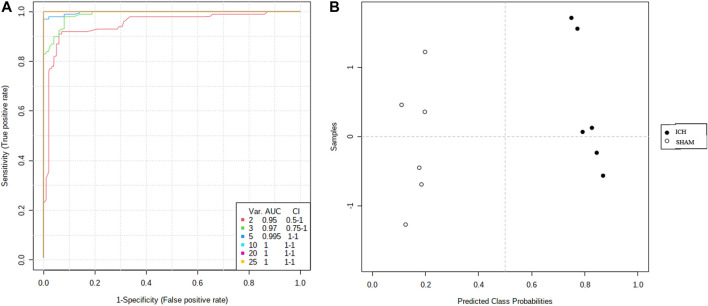
Comparison of the sham and ICH groups of serum samples from multiple ROC curve analysis.

ROC curves were used to evaluate the diagnostic accuracy of the selected serum between ICH and sham subjects. AUC values between 0.7 and 0.9 have “moderate” accuracy, and values greater than 0.9 have “high” accuracy. Figure 8 shows AUC >0.9, sensitivity and specificity are combined to evaluate the clinical diagnostic value of biomarkers have high value.

#### Metabolic Pathway Analysis

**FIGURE 9:**
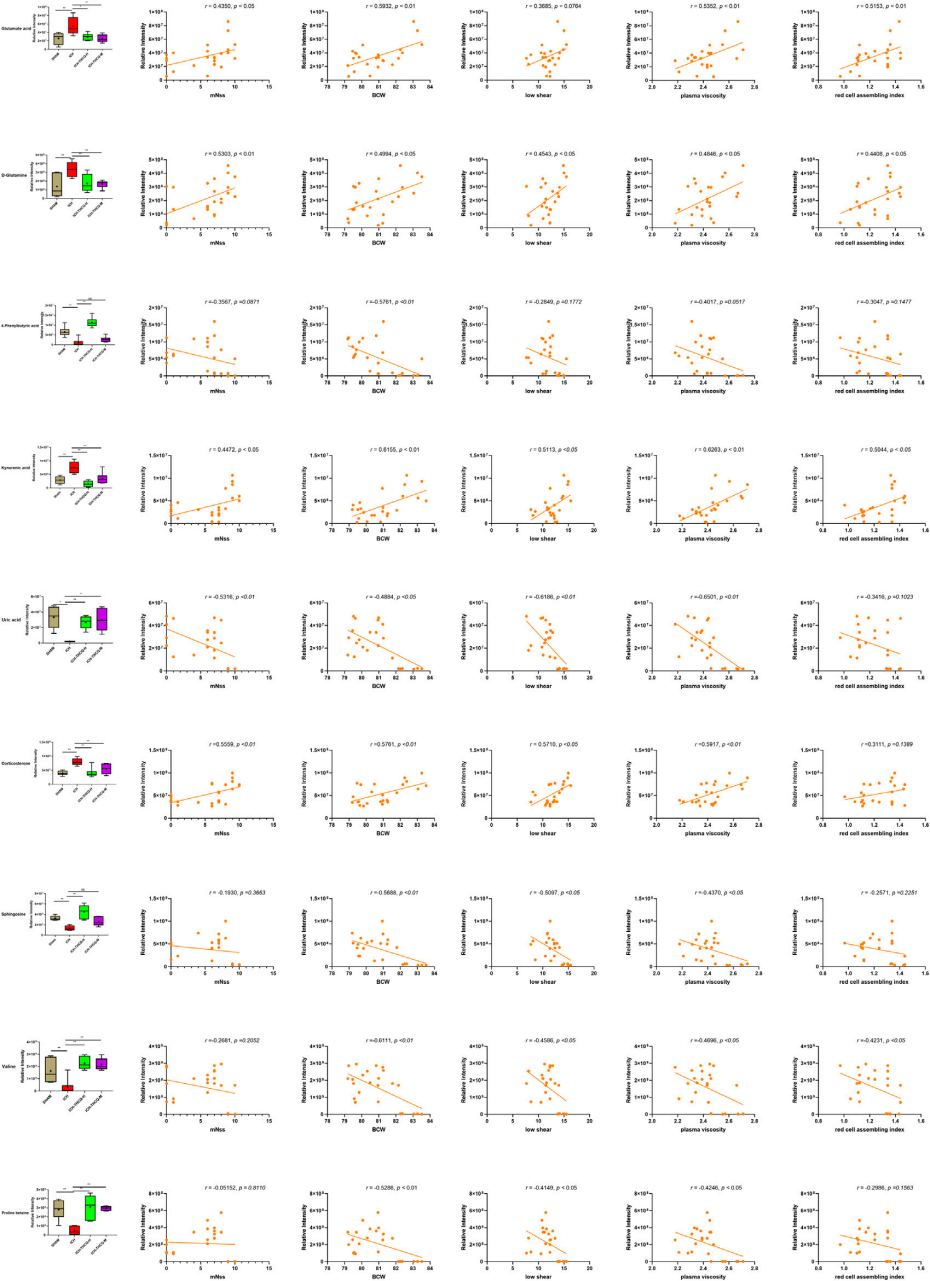
S Serum biomarker levels between different groups **(A)** and their correlations with ICH efficacy indicators **(B**, *mNSS*; **C**, BWC; **D**, LS; **E**, PA; **F**, RCAB**)**, *p < 0.05 and **p < 0.01. The distribution and comparison of serum biomarker levels between different groups **(A)** and their correlations with ICH efficacy indicators **(B**, *mNSS*; **C**, BWC; **D**, LS; **E**, PA; **F**, RCAB**)**, *p < 0.05 and **p < 0.01. **(B)** One metabolite was significantly negatively and four positively correlated with *mNSS* (*p* < 0.05) **(C)** Five metabolites were significantly negatively and four positively correlated with Brain water content (*p* < 0.05). **(D)** Four metabolites were significantly negatively and three positively correlated with Plasma viscosity (*p* < 0.05). **(E)** Four metabolites were significantly negatively and four positively correlated with whole blood aggregation (*p* < 0.05). **(F)** One metabolite was significantly negatively and three positively correlated with whole blood aggregation (*p* < 0.05).

The mechanism of the therapeutic metabolic pathways influenced by THCQ decoction was further explored by inputting the 27 biomarkers listed in Table 1 into MetaboAnalyst (https://www.metaboanalyst.ca/) to conduct pathway analysis. The top 5 enriched metabolic pathways that were mainly affected by THCQ decoction are shown in Supplementary Figure S4, including D-glutamine and D-glutamate metabolism; alanine, aspartate and glutamate metabolism; arginine and proline metabolism; and arginine biosynthesis. Of note, these metabolic pathways are interconnected with each other, and THCQ decoction may affect these different treatment routes to achieve the purpose of treating ICH. Additionally, Spearman correlation was performed to assess the relationship between the metabolic changes involved in D-glutamine and D-glutamate metabolism, sphingolipid metabolism, and glycine, serine and threonine metabolism and the ICH efficacy indicators (*mNSS*, BMC, and hemorheology indexes) (Figure 9).

## Discussion

Intracerebral hemorrhage (ICH) is an acute central nervous system injury resulting in death and disability, and studies of surgical and pharmacological interventions against ICH-induced secondary brain injury (SBI) have been performed in recent decades (Powers et al., 2018). However, secondary brain damage caused by hematoma can lead to severe neurological deficits and death, especially on the first day after the initial attack. A large amount of thrombin is released that causes hematoma enlargement, and this expansion causes edema and nerve destruction, which directly leads to poor prognosis (Keep et al., 2012). Previous studies have shown that persistent bleeding occurs in the hyperacute stage (Broderick et al., 2007). At present, surgical operations mainly focus on hydrocephalus and hematoma removal, but there is great controversy due to the high risk of secondary tissue trauma (Elliott and Smith, 2010). Our research shows that THCQ intervention in the acute phase of ICH can partially block the severe neurological deficit caused by secondary brain injury, reduce blood viscosity and red blood cell accumulation, promote heme efflux, reduce edema and lead to a better prognostic result (Figure 1, Figure 2, and Figure 3).

However, the developmental process and the existence of comorbidities after hemorrhagic stroke are complicated, and little is known about them, with many problems remaining to be resolved. However, we have used many resources and made efforts to clarify the mechanisms involved. However, it was still a huge challenge to understand the occurrence of hemorrhagic stroke at the metabolic level. We also tried to clarify the complex pathophysiological processes from the perspective of metabolomics analysis. In the present study, a total of 27 endogenous metabolites involved in many physiological and functional metabolic pathways were identified in the serum of rats at 72 h after collagenase-induced intracerebral hemorrhage. THCQ decoction partly reversed these alterations. Additionally, in this work, modulation of the metabolome, including 14 amino acids, 7 lipids, purines, quinoline and organic compounds of pyrimidine, pyrimidine, carbonyl compounds, benzene and their substituted derivatives, in the serum of ICH rats was reported for the first time. Understanding the changes in these metabolites may help clarify the mechanism of intracerebral hemorrhage and the therapeutic effects of THCQ decoction.

Our research shows that mitochondrial dysfunction caused by energy metabolism disorders (energy disposal failure, cell apoptosis, etc.), excitotoxicity (Glu-mediated toxicity) caused by amino acid metabolism disorder and redox disorder caused by lipid metabolism disorder (oxidative stress and increased production of reactive oxygen and nitrogen substances) may play an important role in the prognosis of cerebral hemorrhage, causing neuronal damage. THCQ treatment can partially reverse this damage.

### Amino Acid Metabolism and Glutamate-Mediated Excitotoxicity

The results of this work suggest that the levels of 14 amino acids, creatine, L-proline, valine, isoleucine, methionine S-oxide, proline betaine, glutamine, lysine, glutamic acid, methionine sulfoxide, 3-methylhistidine, creatinine, 3-sulfoalanine and N-acetylvaline, significantly change following ICH-induced intracerebral hemorrhage.

The endogenous amino acids valine and isoleucine have protective effects against intracerebral hemorrhage. Valine deficiency is marked by brain nerve function impairment, while isoleucine deficiency is associated with muscle tremor (Málly et al., 1996). In sickle-cell disease, valine is substituted for the hydrophilic amino acid glutamic acid in hemoglobin. The lack of valine in rats can lead to uncoordinated movement, and the lack of valine is a key factor leading to amino acid imbalances and promoting the development of neurotoxicity, which is consistent with previous studies (Hutchison et al., 1983).

After ICH, the levels of valine and isoleucine in the model group decreased, but after THCQ treatment, the levels of both increased, which explained the behavior changes (*mNSS* score) and the reason for the improvement.

The early stage of ICH is characterized by the accumulation of glutamate around the hematoma (Qureshi et al., 2003), and the increase in glutamate levels has an adverse effect on the brain through excitotoxic mechanisms (Babu et al., 2012). Thrombin plays an important role in early bleeding, but with the massive increase in thrombin, red blood cells are lyzed, and glutamate (Glu) accumulates in large amounts in the early stage of experimental hematoma. Glutamate has an effect on the activation of N-methyl-D-aspartate (NMDA) receptors, which may cause a massive influx of calcium to cause irreversible damage while simultaneously stimulating NO production (Rajda et al., 2017). The formation of HO-1 and Fe^2+^ causes oxidative stress and a large increase in neuronal death (Mracsko and Veltkamp, 2014). In the pathological tissue sections, the model group showed an increase in scar formation, inflammatory cell infiltration and cytoplasmic cell proliferation, which fully proved that a series of thrombin-induced injuries occurred (Keep et al., 2012).

Previous reports indicate that glutamine and glutamate in the hippocampus and prefrontal cortex may be important monitoring indicators as cerebrovascular risk factors for stroke (Sun et al., 2014). The increase in serum glutamine in model rats indicates that glutamate may also play an important role in the pathophysiology of cerebral hemorrhage (Sun et al., 2014; Liu et al., 2016).

3-Sulfinoalanine (cysteinesulfinic acid) is an NMDA receptor (Quinn et al., 1997; Stipanuk et al., 2008). The increase in its level may be related to the activation of excitotoxicity by ICH, and the decrease in its level after THCQ administration indicates that THCQ may be able to reverse the resulting toxic injury.

The HMDB database states that 4-phenylbutyric acid can inhibit protein prenylation through the transcriptional activation of the *γ*-globin gene, deplete plasma glutamine, increase hemoglobin production and affect *hPPARγ* activation. At the same time, there is much evidence that *PPARγ* can mediate the scavenger receptor CD36 to affect phagocytosis, clear RBCs from the parenchyma, and increase the expression of antioxidant proteins (Aronowski and Zhao, 2011). Simply put, THCQ-H can stimulate phagocytosis through *PPARγ* and promote the regression and elimination of hematomas, which can reduce inflammation and toxicity and upregulate cellular defense mechanisms (Wu et al., 2018b). In our study, pathway analysis highlights the D-Glutamine and D-glutamate metabolism (Supplementary Figures S4, S5).

### Lipid Metabolism and Antioxidase Deactivation

Previous studies have clearly shown that lipids play important roles in maintaining cell membrane integrity and mitochondrial membrane structure as well as cell signal transmission and energy regulation (Zhang et al., 2020). Our study showed that highly accumulation of lipids was found in the serum of an ICH rat model. However, high-dose THCQ administration increased the levels of 7 intermediates related to lipid metabolism, including carnitine (9-decenoylcarnitine, decanoylcarnitine and stearoylcarnitine), sphingosine, corticosterone, 7-ketodeoxycholic acid, and retinyl acetate. During ICH, lipid synthesis and degradation can be thought to be closely associated with nerve cell damage and repair mechanisms (Hu et al., 2016).

Furthermore, lipid metabolism often participates in the oxidative stress response to cerebral hemorrhage. When the excessive production of OS free radicals (mainly reactive oxygen species (ROS)) exceeds the antioxidant capacity and causes excessive NO production from reactive nitrogen species (RNS), lipids are directly oxidized and participate in the cell death signal transduction pathway to cause cell damage (Sinha et al., 2013). In addition, NO reacts with ö 2 ∙- to form the more toxic compound peroxynitrite (ONOO-), which leads to the oxidation of proteins and the nitration of tyrosine residues (Ding et al., 2014). This has been implicated in neurodegenerative diseases of the central nervous system and stroke (Manzanero et al., 2013; Yan et al., 2013).

According to some reports, sphingosine, corticosterone, retinyl acetate, and 7-ketodeoxycholic acid can participate in the oxidative stress response in cerebral hemorrhage. Among them, sphingosine participates in lipid metabolism disorders by reducing sphingolipid metabolism (Liu et al., 2020). Ceramide is hydrolyzed into sphingosine, which is then phosphorylated by sphingosine kinase to produce S1P. The S1P/S1PR signaling pathway plays an important role in many cellular processes, such as cell survival, migration and cell-to-cell communication (Fyrst and Saba, 2010).

It has been reported that corticosterone induces cell apoptosis (Jiang et al., 2015) and affects spatial memory in rats (Rusu et al., 2018).7-Ketodeoxycholic acid is a bile acid involved in regulating fatty acid metabolism. Bile acids are physiological cleansers that can promote the excretion, absorption and transportation of fats and sterols in the intestines and liver. They modulate both flows of bile and secretion of lipid and are also critical for the absorption of lipid-soluble vitamins and dietary fats. The unique detergent properties of bile acids are critical for the digestion and intestinal absorption of hydrophobic nutrients. Clinically, patients with cerebral hemorrhagic stroke often experience intestinal dysfunction (Li et al., 2017). Intestinal infarction was also observed anatomically in the actual model animal. We speculate that this may be related to the severe lack of bile acid, and THCQ can relieve intestinal infarction after its administration.

Retinol acetate, also known as vitamin A acetate, belongs to a group of organic compounds called retinoids. It can maintain the normal metabolism of the human body, maintain the stability and development of cell membranes, and maintain the normal function of the reproductive system (Aydin et al., 2016). In addition, retinyl acetate is a derivative of retinol, and retinyl is a lipid peroxidation inhibitor (Rajda et al., 2017). Its obvious reduction is a manifestation of the decrease in vitamin levels and the inactivation of antioxidant enzymes, which can be reversed after THCQ administration.

In our study, THCQ decoction administration remarkably restored the serum levels of these lipid intermediates to normal levels. These results indicate that the mechanism by which THCQ decoction relieves ICH is closely related to the regulation of abnormal lipid metabolism.

### Energy Metabolism Disturbance and Mitochondrial Dysfunction

Many perturbed metabolites identified in this study are closely connected to energy metabolism. We observed significant differences in the levels of creatine and decanoylcarnitine between the ICH group and sham groups. Creatine can provide energy for muscles and nerve cells and can also be phosphorylated to form phosphocreatine, which is used for energy storage in the brain (Sun et al., 2014). During SBI, the brain lacks long-term energy storage, so it must transfer a phosphate to ADP from phosphocreatine to form ATP. This phenomenon may be the reason for the significant increase in creatine observed in the model group. Previous studies have reported similar results in cerebrospinal fluid (Braissant et al., 2011).

Decanoylcarnitine is another important metabolite involved in energy metabolism. It is an acylcarnitine. Many diseases that cause disturbances in energy production and intermediate metabolism in organisms have been described and are characterized by unusual levels of acylcarnitine production and excretion (Adeva-Andany et al., 2017). The carnitine shuttle is closely related to the homeostasis of mitochondria and plays an important role in β-oxidation (Adeva-Andany et al., 2017).

Carnitine combines with the deamination products of fatty acids or branched chain amino acids to form acyl carnitine, which transports metabolites into the mitochondria through the cytoplasm and undergoes β-oxidation during degradation to generate energy. Mitochondrial dysfunction leads to decreased ATP synthesis, reduced Ca^2+^ content, and increased levels of ROS and RNS(Beal, 2005). Due to elevated ROS/RNS levels, lipid peroxidation increases, membrane damage occurs, and secondary damage accumulates in mitochondrial DNA (mtDNA) (Rajda et al., 2017). The inability to synthesize ATP will cause the abnormal function of Na^+^/ K^+^ ATPase and damage the sodium-dependent glutamate transporter, leading to increased extracellular glutamate levels and excitotoxicity (Doyle et al., 2008). In addition, energy consumption can also lead to the production of reactive oxygen species and the release of cytochrome c from the outer mitochondrial membrane, leading to cell apoptosis and further brain damage (Babu et al., 2012).

### Other Metabolism Pathways

Kynurenic acid (KYNA) and uric acid (UA) are closely related to the treatment of cerebral hemorrhage. In primates, uric acid is the major antioxidant in serum and is thought to be a major factor involved in lengthening the life span and decreasing age-specific cancer rates in humans and other primates. UA is a physiological end-product of purine metabolism that has strong antioxidant properties. It acts as a water-soluble antioxidant and free radical scavenger in the human body and is related to the prevention and treatment of obesity and stroke (Tang et al., 2019).

KYNA is a quinoline compound that can interact with the glutamate ion-type excitatory amino acid receptor N-methyl-D-aspartic acid (NMDA), α-amino-3-hydroxy-5-methylisoxazole-4-propionic acid, alginate receptors, endogenous antagonists of nicotine, and cholinergic subtype α7 receptors (Plitman et al., 2017; Ramos-Chávez et al., 2018). To date, KYNA is the only identifiable endogenous NMDA receptor antagonist (Plitman et al., 2017). Low concentrations can mediate glutamate-induced hypofunction and promote AMPA receptor activation as well as ODC death and axonal degeneration to protect neurons. A high level of KYNA can lead to psychotic symptoms and cognitive decline, suggesting a two-way adjustment (Rajda et al., 2017). Studies have pointed out that the significant increase in its level in brain diseases is related to the production of free radicals, which can be generated by ONOO^-^(Ramos-Chávez et al., 2018). However, at low concentrations, it can inhibit the release of presynaptic Glu via α7 nicotinic acetylcholine receptors, which makes it an effective neuroprotective agent (Rajda et al., 2015). KYNA expressed by astrocytes can counteract neurotoxins produced by microglia at a local injury site (Füvesi et al., 2012).

UC and 2'-deoxycytidine are pyrimidine compounds, which, along with pyrimidine derivatives, can play important roles in the immune system in processes such as cell adhesion and proliferation (Rajda et al., 2017).

In addition, studies have described thrombin are activated within the first hour after acute ICH (Mracsko and Veltkamp, 2014). With their unprecedented penetration, the coagulation cascade is activated. At the same time, impaired BBB function with subsequent edema formation and neuronal damage at days 1 and 3 after ICH is the characteristic feature in early stages of it. As the lysis of red blood cells leading to the release of hemoglobin, heme oxygenase-1 enzyme (HO-1) is converted into neurotoxic components, which is the main cause of provocative secondary brain injury (Hu et al., 2016; Madangarli et al., 2019). From the actual efficacy and metabolomics exploration, it seems that THCQ decoction has played a certain role in reducing edema, neurotoxicity and anti-oxidation, which is similar to thrombin inhibitors.

Overall, metabolomics analysis showed that THCQ decoction treatment can broadly affect metabolic disorders in ICH rats via multiple metabolic pathways. We note that neurobehavioral scores decreased slightly in the THCQ-H group after 3 days of treatment. The behavior score of stroke patients can reflect the degree of brain damage after cerebral hemorrhage (Han et al., 2018), so the reduction in the behavior score caused by THCQ decoction may be beneficial to inhibit the development of brain damage. However, the behavior scores in the THCQ-M and THCQ-H treatment groups were still very different from those in the sham operation group. The reduction in the behavioral score caused by THCQ decoction was marginal, while the therapeutic effects on improving the blood rheology index and structural changes in brain tissue morphology were more obvious in THCQ decoction-treated ICH rats. Therefore, we are more willing to believe that the neuroprotective effect of the THCQ decoction may occur through promoting clot regression, reducing edema, blocking glutamate-mediated neurotoxicity, and reducing oxidative stress and other regulatory pathways to reduce secondary injury after cerebral hemorrhage. Samples were subjected to metabolomics analysis, serum metabolites whose levels were significantly different were restored by THCQ decoction were mostly distributed in the amino acid metabolism pathway, lipid metabolism pathway, energy metabolic pathway, and uric acid metabolism pathway. THCQ decoction improving the overall metabolism could account for the improvement of brain damage after ICH. In addition, metabolomics is considered to be a useful tool for evaluation of therapeutic agents involved in reducing neuronal damage associated with cerebral hemorrhage.

In addition, it has been reported in previous articles that in cerebral hemorrhage, the P2X7R receptor is a key link between the toxic compound peroxynitrite (ONOO-) and NLRP3. Therefore, we speculate that the traditional Chinese medicine THCQ decoction is likely to work by repairing the upstream and downstream proteases related to ROS and RNS. This is also the focus of our next step in studying the specific mechanisms of the prevention and treatment of ICH.

Moreover, our survey indicated that THCQ decoction treatment at moderate and high does exhibited metabolism-regulating effects to varying degrees. Interestingly, while 4 fewer metabolic biomarkers were identified in the THCQ-M group than in the THCQ-H group, both treatment groups still had 23 biomarkers in common. According to the prognostic results of various previous studies of cerebral hemorrhage, it was initially determined that moderate and high doses of THCQ decoction were more effective than low doses. Therefore, the metabolome study was only performed for the moderate and high doses. Studies have shown that high doses can regulate more metabolites and better activate its therapeutic effectors on metabolic disorders. Nevertheless, THCQ decoction can effectively alleviate the neuropathological changes and improve the behavior of ICH rats of two doses. However, this is the primary test using an intracerebral hemorrhage rodent model with a relatively small sample size. More trials comparing different THCQ decoction combinations, doses, and durations and further research on toxicity and adverse effects are needed.

In this study, we chose to establish the linkage between the metabolic and neuroprotective effects of THCQ decoction. It is a challenging subject in that there exists little to no consensus. However, some metabolic biomarkers found in our research have been reported to be related to cerebral hemorrhage. Therefore, correlation trend analysis may help us to explore the correlation between biomarkers and ICH. As mentioned above, various metabolic pathways regulated by THCQ decoction, such as amino acid metabolism and lipid metabolism, as it has already been reported to be related to the development of cell excitotoxicity and oxidative stress in ICH. In addition, correlation analyses were performed between metabolites involved in amino acid metabolism/lipid metabolism and five most sensitive index of the physiological condition and damages to the brain nerve. [neurobehavioral score, BWC, low shear, PV and red cell aggregation index (RCA)], our results showed that serum levels of these metabolites had a certain correlation with the ICH-related indicators. Taken together, the metabolic pathways regulated by THCQ decoction were related to ICH development, and some of them were correlated with indicators of neuronal damage. It was suggested that THCQ decoction may contribute to neuroprotection in ICH rats via the overall regulation of metabolic disorders.

## Conclusion

We validated that treatment with an appropriate dose of THCQ decoction had neuroprotective effects in ICH models. Furthermore, metabolomics studies have shown that THCQ decoction treatment could significantly improve the levels of abnormal metabolites related to glutamate-mediated excitotoxicity, antioxidant deactivation, mitochondrial homeostasis/dysfunction, and other metabolic processes closely related to the progression of ICH. THCQ decoction acts as a thrombin activity inhibitor. It can inhibit the production of excessive thrombin during the entry of blood into the brain parenchyma after ICH, reduce early edema, remove oxidative metabolites, reduce cell death by removing endogenous toxic substances, and stabilize the energy support of nerve cells by reducing the excitotoxicity of glutamate and the damage of neurons by oxidative stress. In addition, there was a clear correlation between the indicators of brain nerve damage and the serum levels of metabolic biomarkers. These mechanisms may collectively contribute to the enhancement of the neuroprotective effect of THCQ decoction on ICH-induced metabolic disorders.

## Data Availability

The original contributions presented in the study are included in the article/Supplementary Material, further inquiries can be directed to the corresponding author.
