# Supplementary figures and images for: Metabolic Insight Into the Neuroprotective Effect of Tao-He-Cheng-Qi (THCQ) Decoction on ICH Rats Using Untargeted Metabolomics
Source: Front Pharmacol. 2021 May 3;12:636457. doi: 10.3389/fphar.2021.636457 (PMC8126979; doi:10.3389/fphar.2021.636457)

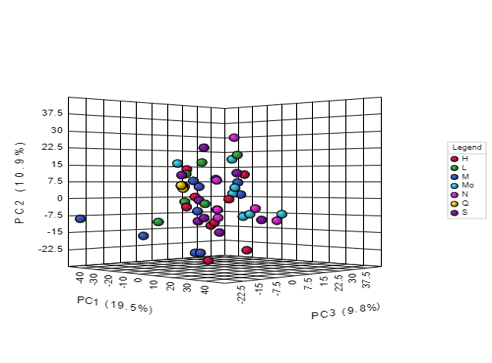

Supplement: Supplementary file 2 [file image3.tif]

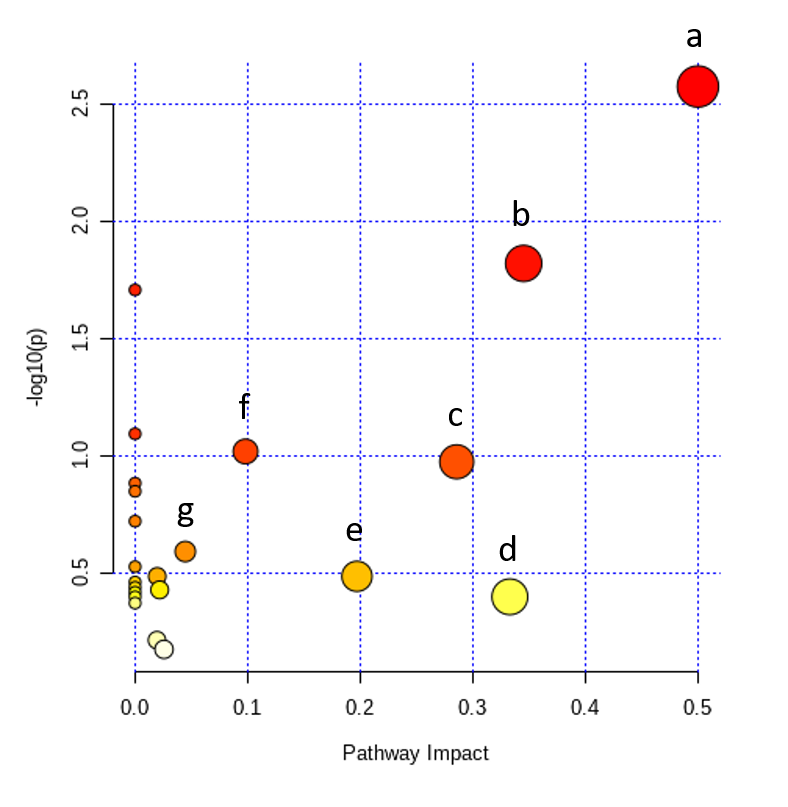

Supplement: Supplementary file 3 [file image4.tif]

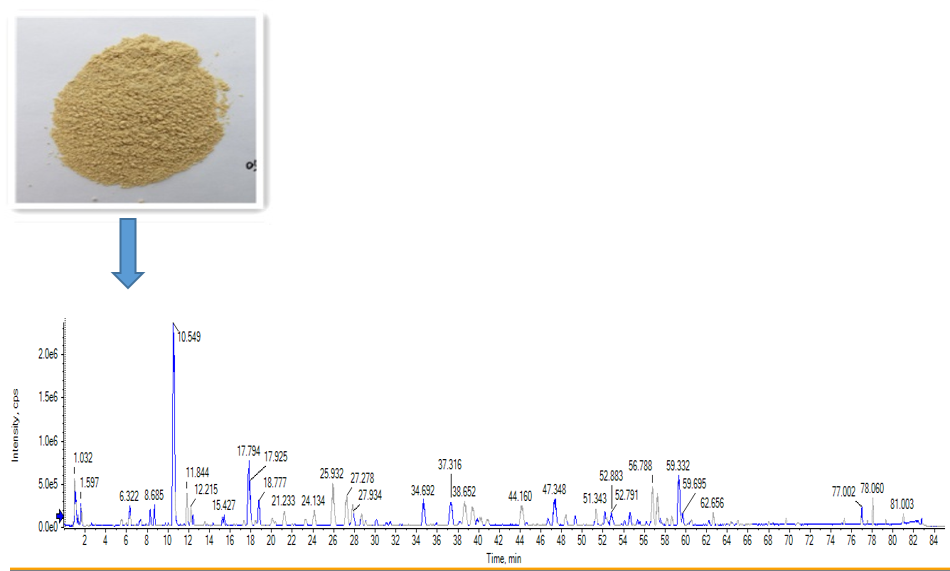

Supplement: Supplementary file 5 [file image2.tif]

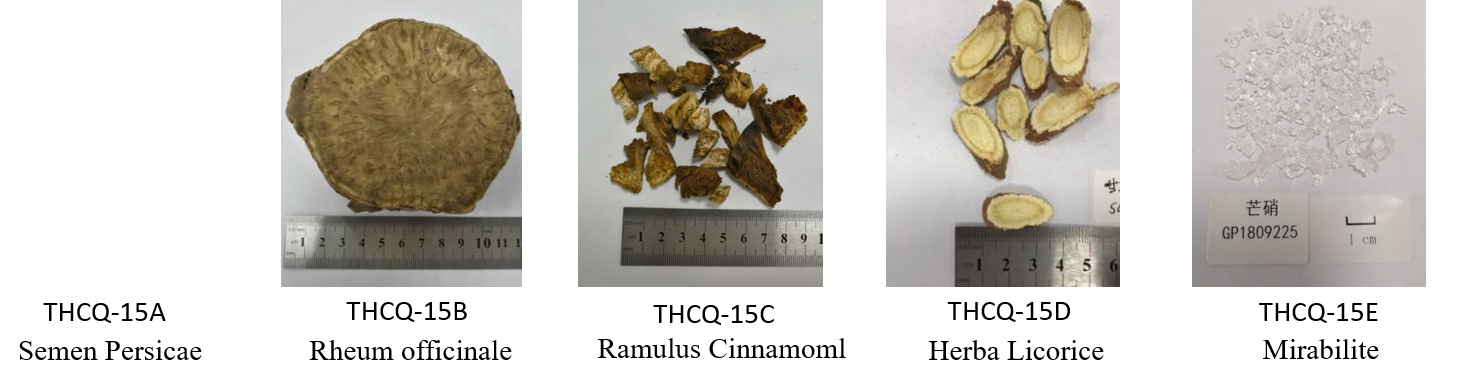

Supplement: Supplementary file 7 [file image1.tif]

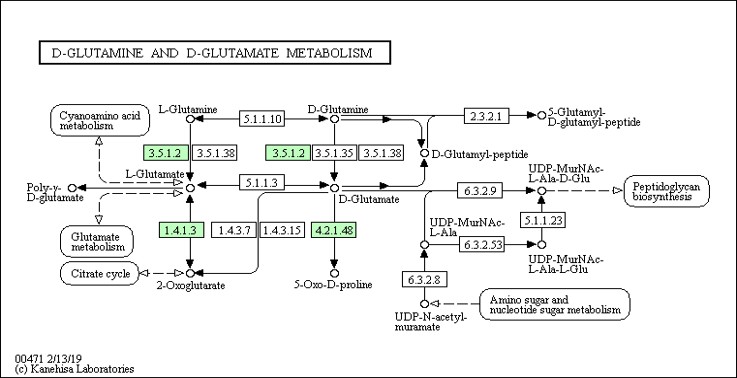

Supplement: Supplementary file 10 [file image5.tif]
